# Time-resolved map of serum metabolome profiling in D-galactose-induced aging rats with exercise intervention

**DOI:** 10.1016/j.isci.2024.108999

**Published:** 2024-01-26

**Authors:** Xue Li, Changling Wei, Yu Jin, Jinmei Zhang, Pei Zhong, Deman Zhang, Xiaohan Huang

**Affiliations:** 1 School of Sports Medicine and Health, Chengdu Sport University, Chengdu, Sichuan Province 610041, China; 2 iCarbonX Diagnostics (Zhuhai) Company Limited, Zhuhai, Guangdong Province 518110, China

**Keywords:** Biological sciences, Physiology, Omics, Metabolomics

## Abstract

Exercise, an intervention with wide-ranging effects on the whole body, has been shown to delay aging. Due to aging and exercise as modulator of metabolism, a picture of how exercise delayed D-galactose (D-gal)-induced aging in a time-resolved manner was presented in this paper. The mapping of molecular changes in response to exercise has become increasingly accessible with the development of omics techniques. To explore the dynamic changes during exercise, the serum of rats and D-gal-induced aging rats before, during, and after exercise was analyzed by untargeted metabolomics. The variation of metabolites was monitored to reveal the specific response to D-gal-induced senescence and exercise in multiple pathways, especially the basal amino acid metabolism, including glycine serine and threonine metabolism, cysteine and methionine metabolism, and tryptophan metabolism. The homeostasis was disturbed by D-gal and maintained by exercise. The paper was expected to provide a theoretical basis for the study of anti-aging exercise.

## Highlights

• Exercise maintained the metabolism homeostasis, which was disturbed by D-galactose
• Trends of metabolites were monitored during exercise to draw time-resolved map
• Changes of metabolites were detected at 4 weeks of recovery after exercise

## Graphical abstract

**Figure:**
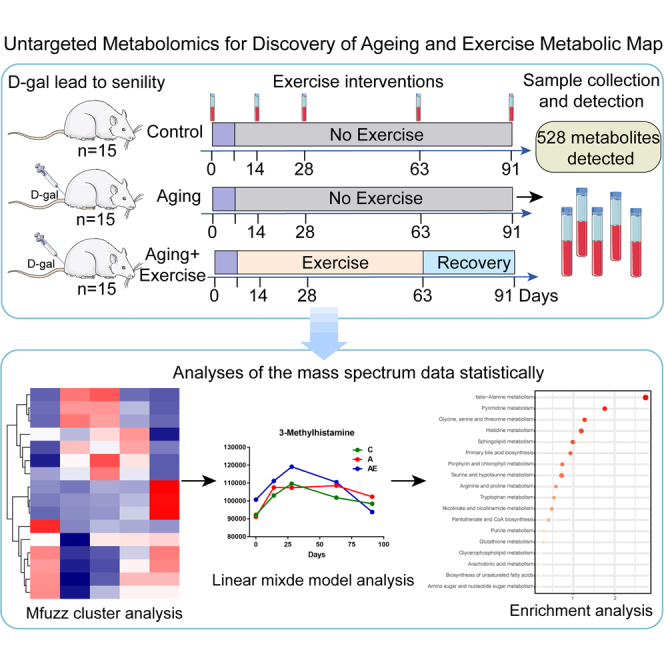

## Introduction

With the increase of human life expectancy, declined cognitive competence and memory impairment threaten the quality of life,^1^ which is becoming a severe social problem.^2^ Interventions have been searched to delay the onset of cognitive impairment in aging time. However, pharmacological interventions have been proven to be far less effective than expected; the behavioral interventions then received widespread attention.^3^ Exercise can extend life expectancy as a non-pharmacological tool by maintaining physical fitness and improving cardiovascular and neurological function.^4^^,^^5^ Studies showed that various active substances released by physical exercise contributed to improving age-related neurodegenerative diseases.^6^ For example, brain-derived neurotrophic factor (BDNF) has been proven to regulate the effect of exercise on synaptic plasticity and cognitive function.^5^^,^^7^^,^^8^ Moreover, Yang and Kwon found that myokines released from tissues during physical activity can promote physical health. In addition, the levels of various metabolites were changed during exercise.^9^ Both exercise and aging were the regulators of systemic metabolism.^10^ Metabolic dysregulation is one of the hallmarks of aging.^11^ Therefore, it is particularly important to monitor the variety of metabolism in the aged with exercise intervention.

Aging is a complex process characterized by a gradual decline of cellular and organ function and is regulated by a variety of factors such as genetics, environment, diet, and lifestyle. Although both cumulative oxidative stress and DNA damage contribute to aging, progressive metabolic dysfunction is a more common marker of biological aging.^12^ From a metabolic perspective, the aging hypothesis includes the mitochondrial and calorie restriction hypothesis.^13^ The mitochondrial hypothesis, proposed by Harman, is also known as the oxidative stress hypothesis because more than 90% of oxidative stress is attributed to mitochondria.^13^^,^^14^ The hypothesis suggests that oxidative stress is one of the causes of aging; thus the increase in antioxidants can significantly extend lifespan, such as glutathione. The calorie restriction hypothesis suggests that reduced calorie intake is accompanied by extended life expectancy.^13^^,^^15^ During calorie restriction, oxidative stress is reduced consistently and various signaling responses, such as sirtuin and nicotinamide mononucleotide, was activated.^13^^,^^16^ In experimental models, some compounds or metabolites exert life-extending effects, such as nicotinamide mononucleotide and α-ketoglutarate.^17^ Moreover, metabolites involving nicotinamide adenine dinucleotide, α-ketoglutarate, reduced nicotinamide dinucleotide phosphate, and β-hydroxybutyrate were regarded as the central mediator of aging, whose levels decline with aging.^11^ The complementary of the aforementioned metabolites increased life expectancy and improved symptoms associated with aging.^11^ Metabolic disorders during aging are particularly pronounced in the central nervous system due to the enormous energy demands of the brain.^12^ The brain depends mainly on the metabolism of glucose, ketone bodies, and amino acids.^18^ Several metabolites in the cerebrospinal fluid were identified by Lin and his colleagues as significantly associated with the aging process in the cerebral circulation. Compared with younger adults, the levels of alanine, citrate, creatinine, lactate, leucine, tyrosine, and valine in old were increased significantly, implying increased anaerobic glycolysis, mitochondrial dysfunction, and reduced glucose utilization in the cerebral circulation.^12^ These suggest that aging is closely related to metabolic dysregulation.

Exercise is beneficial to the quality of life in the old, which prevents age-related diseases and slows the age-related decline in physical function by mitigating potentially harmful oxidative damage and suppressing inflammatory processes.^19^ During the exercise period, skeletal muscle, an important secretory organ, experienced the most significant remodeling, which delivered the metabolic stress to distant tissues including the central nervous system by secreting the myokines.^5^^,^^20^ Myokines had been shown to improve glucose treatment and regulate the metabolism of glucose and lipid by increasing the sensitivity to insulin.^21^ One of the most observed myokines was BDNF, which played a role in both central and peripheral metabolism and had been identified as a shrinkage-induced protein in skeletal muscle capable of enhancing lipid oxidation in skeletal muscle by activating adenosine monophosphate-activated protein kinase (AMPK).^5^^,^^7^^,^^22^ A similar finding showed increased polyunsaturated free fatty acids, decreased ceramides, sphingomyelin, and phospholipids, as well as changes in gut microbiome metabolites and redox homeostasis, during exercise. Moreover, aging is accompanied by a loss of skeletal muscle mass and function (sarcopenia).^23^ The main differences in skeletal muscle metabolite levels between young and healthy elderly subjects were related to mitochondrial function, muscle fiber type, and tissue renewal. Prolonged resistance-type exercise training led to an adaptive response to amino acid metabolism, which was reflected by decreased catabolism of branched-chain amino acids in the old.^24^ Thus, the combination of branched-chain amino acid intake and exercise therapy significantly improved total lower extremity muscle strength and the ability to dynamic balance in the old.^25^

Although researchers had provided evidence that exercise acts as an effective strategy to delay aging, most current studies focused on the changes in metabolites before and after exercise and lacked changes during exercise. The mechanisms of how exercise benefits older individuals are still not fully understood. To fill this knowledge gap and provide a comprehensive resource on the dynamic changes in metabolite pathways of exercise to delay aging, we performed untargeted metabolomics analysis on serum before, during, and after exercise in aging and exercising rats, to monitor changes in metabolites over time. In this paper, D-galactose (D-gal)-induced aging model was used to simulate the process of aging, which was widely used for pharmacodynamic evaluation and researching the mechanism of aging and aging-related cognitive impairment.^26^

## Results

### Exercise alleviated the damage of spatial learning and memory ability induced by D-gal in rats

**Figure 1:**
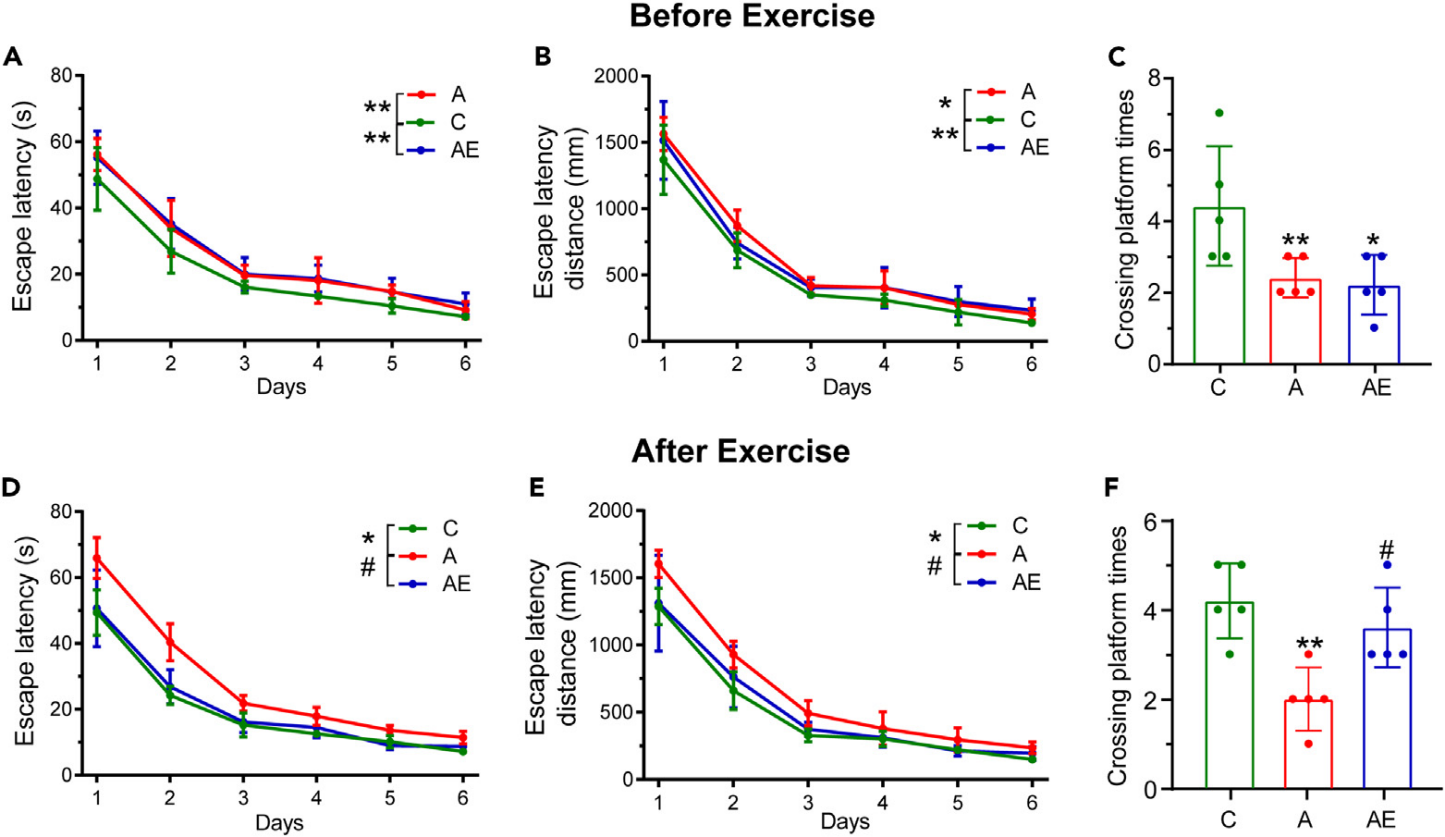
Exercise alleviated the spatial learning and memory ability of rats damaged by D-gal, which were detected by MWM test (A), (B), and (C) were tested before exercise intervention. (D), (E), and (F) were tested after exercise intervention (n = 5, Data were represented as mean ± standard deviation and were analyzed by one-way ANOVA analysis of GraphPad Prism 9. ∗∗p < 0.01 and ∗p < 0.05 indicate significant difference compared with C group, respectively; #p < 0.05 indicates a significant difference compared with A group. The comparison of (A), (B), (D), and (E) was done with the average of data for the entire period during the navigation phase.(A) and (D) Escape latency of rats on 1st–6th days in the navigation phase (B) and (E) Escape latency distance of rats on 1st–6th days in the navigation phase (C) and (F) The times of crossing platform of rats on the 7th day in the spatial exploration phase. See also Figure S1.

Rats were injected with D-gal for 6 weeks, which significantly induced senescence in rats, resulting in impaired spatial learning and memory abilities (Figures 1A–1C). During the navigation phase, the escape latency and escape latency distance of rats in group A (D-gal-induced aging) were significantly higher than those in group C (normal control) (Figures 1A and 1B). The times of crossing platform during the spatial exploration phase were also significantly less than those in group C (Figure 1C), indicating the successful establishment of the aging model. Meanwhile, after the injection of D-gal, the D-gal rats were characterized with lighter body weight, reduced superoxide dismutase (SOD) level and, and elevated malondialdehyde (MDA) in serum (Figures S1A, S1C, and S1D). After the exercise intervention for 56 days, the Morris water maze (MWM) was tested again, of which results showed that the escape latency and escape latency distance of group A were significantly higher than those of group C and AE (D-gal-induced aging plus exercise) during the navigation phase (Figures 1D and 1E). Similarly, the times of crossing platform of group AE were also significantly higher than those of group A (Figure 1F), which indicated that exercise significantly alleviated the D-gal-induced impairment of spatial learning and memory ability in aging rats. Moreover, at the end of exercise, the body weights of group AE were slightly higher than those of group A (Figure S1B). The levels of MDA and SOD in rats were significantly changed after exercise (Figures S1C and S1D), which indicated that exercise delayed the aging process.

### Regulation of the serum metabolites in response to D-gal-induced aging and exercise

**Figure 2:**
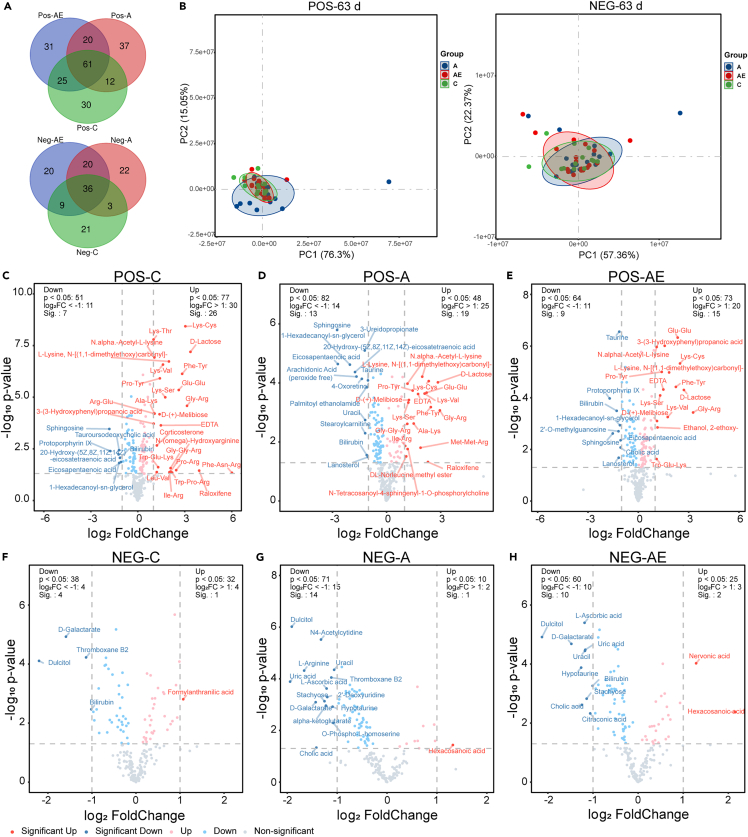
Exercise and aging regulated the homeostasis of serum metabolites in rats (A) Venn diagram of metabolites with significant change compared the 63rd day with the 0th day. (B) PCA of C, A, and AE groups on the fourth 63rd day (confidence interval = 0.9). (C), (D), and (E) were the volcano plots metabolites in the positive ion model. (F), (G), and (H) were the volcano plots metabolites in the negative ion model. The horizontal dashed line represents the threshold for p value of 0.05, and the vertical dashed lines represent thresholds for ± 2-fold changes. Both the 63rd day and the 0th day of the three groups were analyzed using paired tests. See also Figure S2.

To understand the variable trends of metabolites over time, the blood samples were collected before exercise (the 0th day), during exercise (the 14th day, 28th days, and 63rd day), and after exercise (the 91st day). The blood samples were detected by an untargeted metabolomics approach and analyzed by a self-developed metabolomics analysis platform. And the metabolite identification was based on autonomous construction. The stability of the experiment was judged by the quality control (QC) sample. The total ion chromatography (TIC), principal-component analysis (PCA), and coefficient of variation (CV) suggested the accuracy and stability of test results (Figure S2). 326 metabolites were found in the positive ion data (POS) and 202 metabolites in the negative ion data (NEG) after preprocessing of the metabolic data. The expression of metabolites on the 63rd day was compared with that on the 0th day of three groups and was analyzed with paired tests, which showed that the 128, 130, and 137 positive metabolites changed significantly (p < 0.05) over time in group C, A, and AE, respectively. And in the negative ion mode, 69, 81, and 85 metabolites changed significantly (p < 0.05) over time in groups C, A, and AE, respectively. Moreover, 61 positive and 36 negative metabolites were changed significantly in three groups (Figure 2A). But the PCA revealed that variations in total analytic metabolites mainly accounted for individual differences, with clear separation along with exercise and aging (Figure 2B). Further data analysis, including Mfuzz cluster analysis and enrichment analysis, was used to identify the pathways of exercise-regulated aging. Time-resolved regulation is an indication of the dynamics of metabolites.^27^ Consequently, linear mixed model (LMM) analysis was used to determine the model of metabolites changed over time, which provided important clues for understanding the changes in metabolite levels during exercise.

In the span of intervention time, almost all detected metabolites changed. A significant change was observed in 38, 47, and 36 metabolites in groups C, A, and AE, respectively, which were screened by adding the absolute value of Log_2_ fold change > 1 based on the aforementioned differential metabolites. Among these, 13 and 5 metabolites were specific to the A and AE groups, respectively. Significant changes in metabolites during exercise reflect the consumption of substrates (such as amino acids and peptides). Peptides such as Glu-Glu, Gly-Arg, Lys-Cys, Lys-Ser, Lys-Val, Phe-Tyr, and Pro-Tyr were increased significantly over time in all three groups. Interestingly, 3-ureidopropionate, 4-Oxoretinol, arachidonic acid, palmitoyl ethanolamide, uracil, 2′-deoxyuridine, α-ketoglutarate, L-arginine, N4-acetylcytidine, and O-phospho-L-homoserine were decreased significantly and DL-norleucine methyl ester, Met-Met-Arg, and N-tetracosanoyl-4-sphingenyl-1-*O*-phosphorylcholine were increased significantly over time only in group A (Figures 2D and 2G). The aforementioned results indicated that metabolism disorders occurred in D-gal-induced aging individuals, while exercise coordinated metabolism back to normal. Specifically, 3-(3-Hydroxyphenyl)propanoic acid and Trp-Glu-Lys were increased significantly while bilirubin and protoporphyrin IX were reduced significantly over time in both C and AE groups (Figures 2C, 2E, 2F, and 2H), which reflected that exercise modulated the changes in metabolites of D-gal-induced aging individuals more prone to youth (Figure 2).

### Cluster analysis reveals similar and different metabolites in D-gal-induced aging and exercise

**Figure 3:**
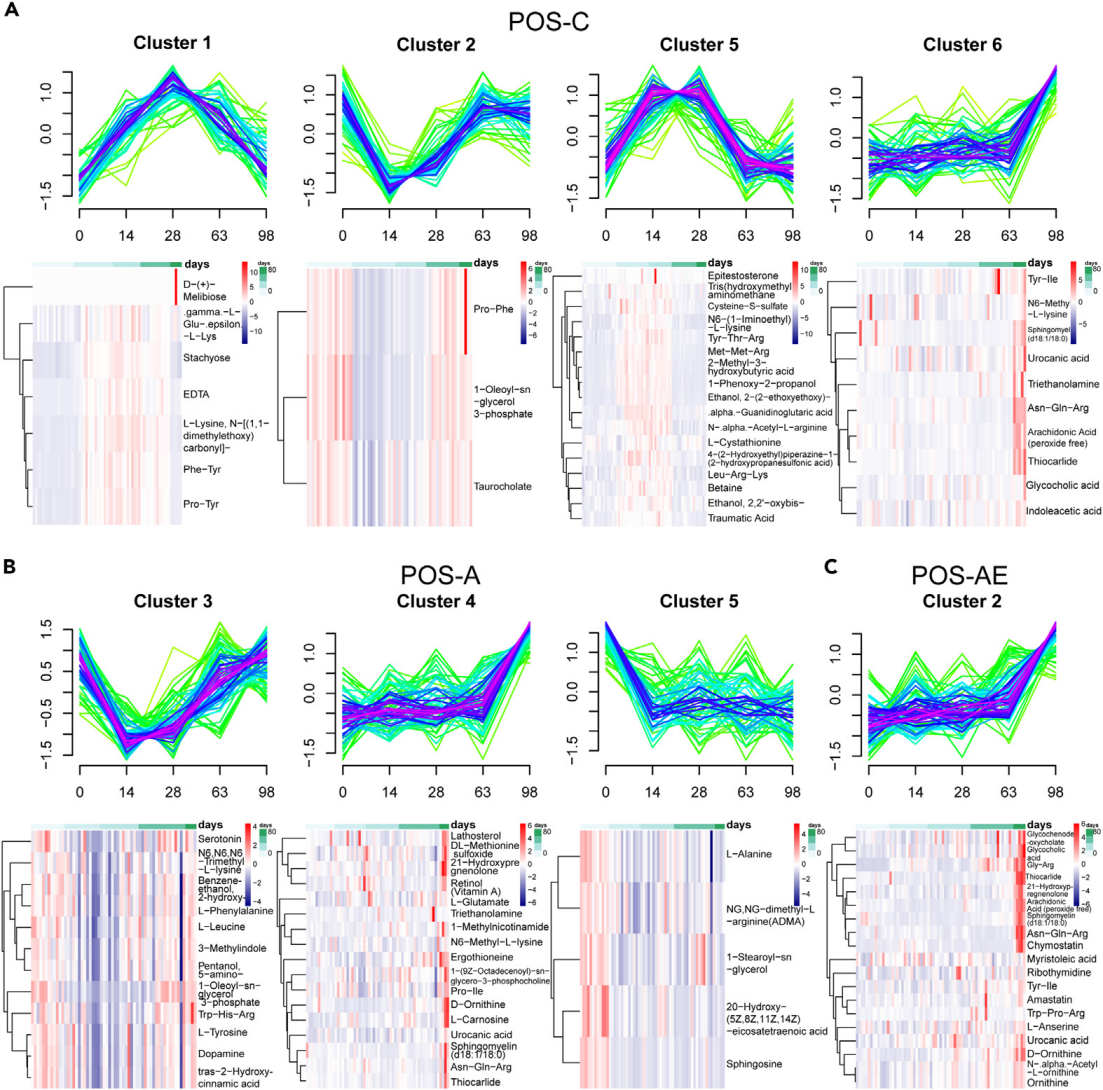
Cluster analysis reveals similar and different metabolites in aging and exercise (A), (B), and (C) were analysis of individual behavior and pattern of metabolite (positive). (A), (B), and (C) were C, A, and AE groups, respectively. See also Figure S3.

**Figure 4:**
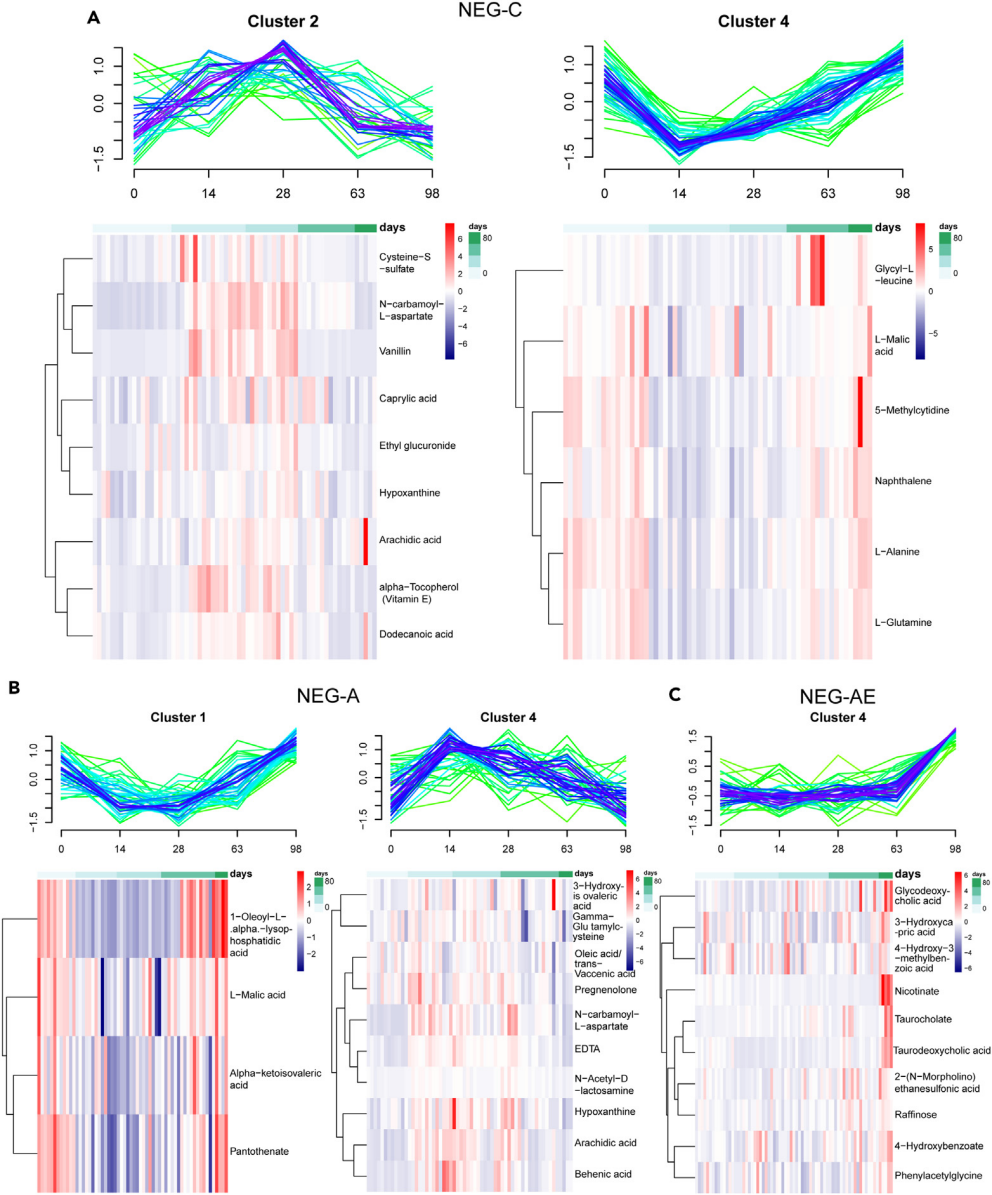
Analysis of individual behavior and cluster of metabolite (negative) (A), (B), and (C) were the C, A, and AE groups, respectively. See also Figure S4.

According to minimum centroid distance, the optimal number of clusters of C, A, and AE groups was 6, 5, and 5 (Figure S3), respectively. And the metabolites with negative iron were stratified into 13 clusters with 4, 4, and 5 of those to groups C, A, and AE, respectively (Figure S4). In each cluster, the metabolite with a membership score > 0.7 was selected to draw the heatmap. It was found that the performance of these metabolites on individuals was consistent with the pattern (Figures 3 and 4). In positive iron, cluster 1, cluster 2, cluster 5, and cluster 6 of group C had core metabolites. Cluster 3, cluster 4, and cluster 5 in group A and cluster 2 in group AE had core metabolites (Figure 3). Meanwhile, clusters 2 and 4 (NEG-C), cluster 1 and 4 (NEG-A), and cluster 4 (NEG-AE) had core metabolites (Figure 4).

Cluster 1 and cluster 5 of POS-C, cluster 2 (NEG-C), and cluster 4 (NEG-A) all demonstrated an initial upregulation followed by a downregulation. The peak of the curve usually occurred on the 14th and 28th day. Reversely, clusters 2 (POS-C), cluster 3 and cluster 5 (POS-A), cluster 4 (NEG-C), and cluster 1 (NEG-A) signified a cluster of downregulation with a slow return. The bottom end of the falling curve was also on the 14th and 28th day. Interestingly, cluster 2 (POS-AE), cluster 4 (NEG-AE), cluster 6 (POS-C), and cluster 4 (POS-A) showed a constant or maintained upregulation over time. The upregulation was slow during exercise while that was rapid in the recovery phase (Figures 3 and 4). Of note, with the exercise intervention, most metabolites tended to increase gradually over the course of the exercise and remained on the rise after the recovery phase, while the metabolites were likely to shift at intermediate time points.

**Figure 5:**
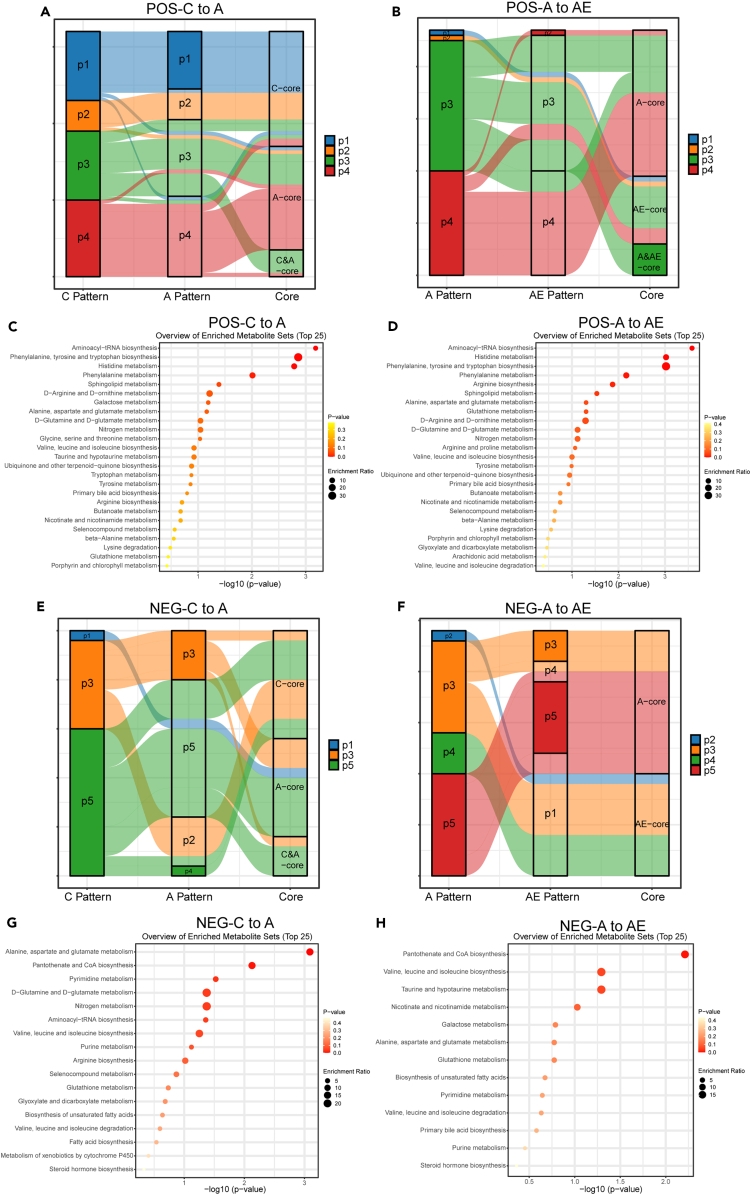
Changes in the pattern of core metabolites and the enrichment of metabolites changed in the pattern of the core (A) and (E) The changes in patterns and the cluster score of positive and negative metabolites compared C with A group, respectively. (B) and (F) The changes in patterns and the cluster score of positive and negative metabolites compared A with AE group, respectively (The different colors indicate the pattern of the metabolites over time. The “core” in the third column indicates the metabolites with membership score > 0.7.). (C) and (G) The enrichment of positive and negative metabolites changed in patterns and the cluster score compared C with A group, respectively. (D) and (H) The enrichment of positive and negative metabolites changed in patterns and the cluster score compared A with the AE group, respectively. See also Figure S5 and Table S1.

To compare whether the clusters of metabolites changed over time, among the different groups, the 16 clusters (POS) and 13 clusters (NEG) of three groups were divided into 4 and 5 broad categories, respectively: pattern 1, pattern 2, pattern 3, pattern 4, and pattern 5 in order (Figure S5). Compared to C with A group and A with the AE group, the changes of core metabolites were shown in Figure 5. In the C and A groups, there were 64 core positive metabolites, 6 of which were with changed pattern. Compared to A with AE group, there were 47 core metabolites in total, 10 of which were with changed pattern. The others retained the same pattern, but the score of clusters changed (Figures 5A, 5B, and Table S1). The aforementioned changes were similar to the metabolites in negative. There were 25 core metabolites in groups C and A, the patterns of 7 of which were changed. In groups A and AE, there were 24 core metabolites in total, the pattern of 14 of which were changed. The others retained the same pattern, but the score of clusters changed (Figures 5E, 5F, and Table S1).

In a comparison of group C, metabolites with altered clusters and patterns in group A were mainly enriched in the following pathways: phenylalanine, tyrosine, and tryptophan biosynthesis; histidine metabolism; alanine, aspartate, and glutamate metabolism; pantothenate and coenzyme A (CoA) biosynthesis; sphingolipid metabolism; pyrimidine metabolism; and D-amino acid metabolism. Similarly, histidine metabolism; phenylalanine, tyrosine, and tryptophan biosynthesis; sphingolipid metabolism; and pantothenate and CoA biosynthesis were altered by exercise. However, the pathways of arginine biosynthesis; valine, leucine, and isoleucine biosynthesis; alanine, aspartate, and glutamate metabolism; and taurine and hypotaurine metabolism were special for exercise (Figures 5C, 5D, 5G, and 5H). It indicated that the amino acid metabolism was regulated by D-gal-induced aging and exercise, which specifically activated the synthesis of amino acid and their derivatives.

### Metabolic pathways regulated by D-gal-induced aging and exercise

In Figure 2A, comparing the 0th day with the 63rd day, hundreds of metabolites were observed that changed significantly. And it has been found that the variation tendency of metabolites was regulated by D-gal-induced aging and exercise in the aforementioned results. To further determine the trend of changes, these metabolites were analyzed by LMM. To verify whether the patterns of metabolites changed over time, patterns were classified into four categories (model1 up, model1 down, model2 up, and model2 down) based on the order of the variable in the LMM and the positive or negative coefficients of the variable (Figure S6).

**Figure 6:**
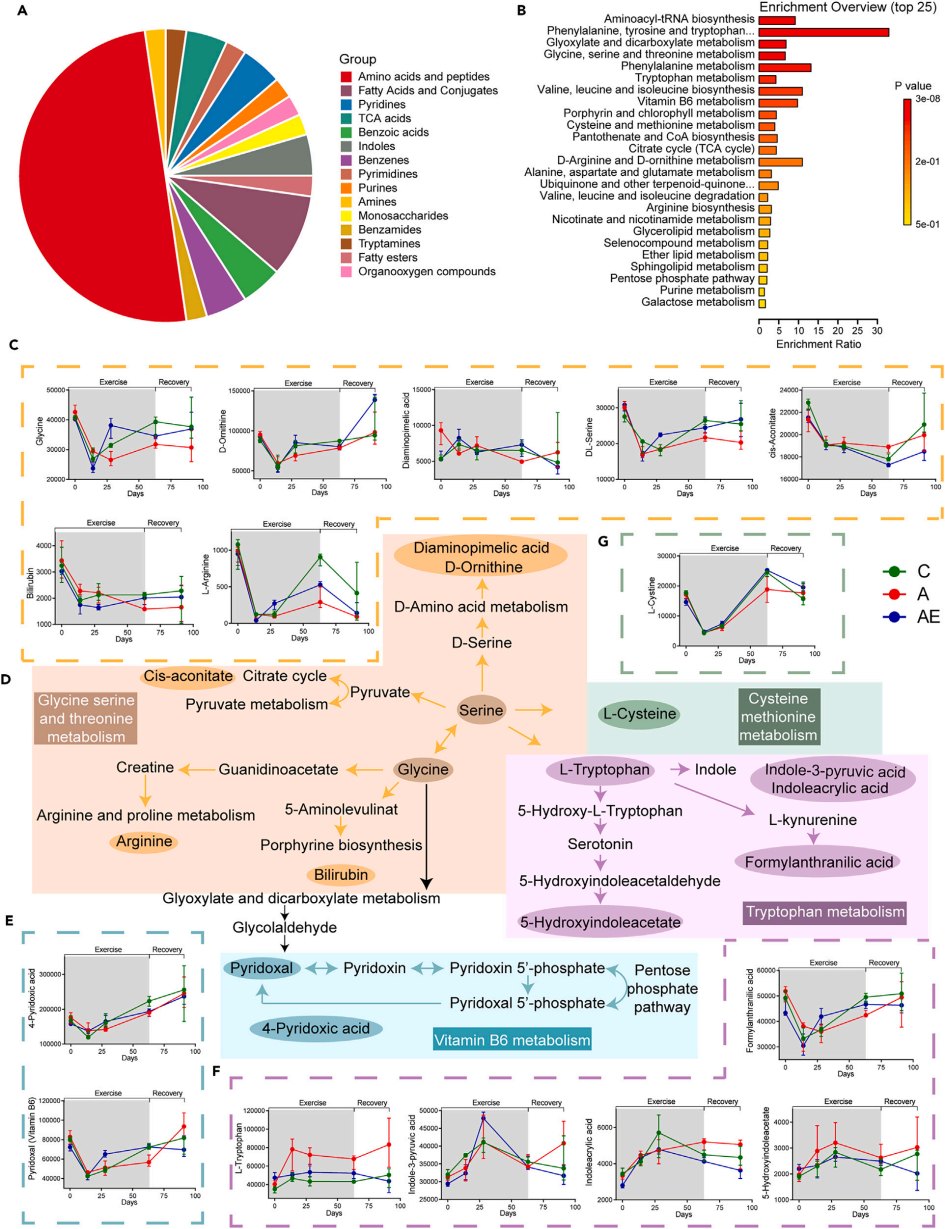
Metabolic pathways regulated by aging and exercise (A) The main class of metabolites. (B) The KEGG enrichment of metabolites with the same trend between groups C and AE and that was opposite to group A. (C) The metabolites of glycine serine and threonine metabolism. (D) Schematic representation of selected metabolism of glycine, serine, and threonine metabolism; cysteine and methionine metabolism; tryptophan metabolism; and vitamin B6 metabolism. (E) The metabolites of vitamin B6 metabolism. (F) The metabolites of tryptophan metabolism. (G) The metabolites of cysteine methionine metabolism (Data of (C), (E), (F), and (G) were represented as median ± interquartile range). See also Figures S6, S7 Tables S2 and S3.

The statistical result of metabolites changed significantly over time in four models showing that most metabolites increased or decreased in model 2 rather than model 1, which indicated that most metabolites did not increase or decrease with a straight line. Moreover, the number in each model of group AE was similar to that in group C, which was different from that in group A (Table S2). Metabolites with the same trend between groups C and AE and that was opposite to group A were selected for Kyoto Encyclopedia of Genes and Genomes (KEGG) enrichment. The top five categories of them with the highest percentage were amino acids and peptides, fatty acid and conjugate, pyridines, tricarboxylic acid (TCA), and benzoic acid in that order (Figure 6A). Meanwhile, the metabolites were enriched in the metabolism, including kinds of amino acid metabolism and biosynthesis, vitamin B6 metabolism, porphyrin and chlorophyll metabolites, pantothenate and CoA biosynthesis and TCA cycle, etc. (Figure 6B). The aforementioned results indicated that the expression and metabolism of amino and peptides were disrupted by D-gal-induced aging while those were recovered by exercise leading to being similar to the youth (group C).

31 metabolites were selected by consulting the literature related to aging closely and performed on the pathway analysis (Table S3, and Figure S7), including glycine, serine, arginine, and tryptophan, etc. The major metabolites and metabolic pathways were plotted in Figures 6C–6G combined with the KEGG analysis, including glycine, serine, and threonine metabolism; tryptophan metabolism; cysteine methionine metabolism; and vitamin B6 metabolism. Most of the metabolites were amino acids and their derivatives, which did not increase or decrease with time singularly. The first inflection point was prone to occur in the 14th or 28th day to reverse the change trend. The second inflection point was usually at the end of exercise when metabolite levels rose or fell sharply (Figures 6C, 6E, 6F, and 6G). Most metabolites involved in glycine, serine, and threonine metabolism; cysteine methionine metabolism; and vitamin B6 metabolism showed a downward trend followed by a plateauing or increasing trend (Figures 6C, 6E, and 6G), which indicated exercise accelerated the production of those metabolites. Conversely, most metabolites involved in tryptophan metabolism showed an increasing trend followed by a plateauing or decreasing trend (Figure 6F), indicating that exercise enhanced the utilization of that metabolites. D-gal-induced aging disturbed the basal amino acid metabolism, which recovered from exercise to certain extent to maintain balance. Thus, we hypothesized that exercise improved aging organisms by regulating essential amino acid metabolism in D-gal-induced aging individuals.

## Discussion

The aging model was established with D-gal, whose learning and memory ability were assessed by MWM. Compared with natural aging model, the D-gal aging model had shorter modeling time, more convenient operation, fewer side effects, and higher survival rate throughout the trial.^28^^,^^29^ The anti-aging benefits of exercise have been generally accepted for a long time ago. However, the dynamic changes of the metabolites over time remain incompletely understood. Most research works had a relatively small sample size and limited duration, and not all studies compared the differences in biomarkers between the control and experimental groups.^30^ With the development of technology, it is possible to fully map the biochemical pathways involved in the movement.^27^ In this paper, we used untargeted omics assays to monitor the time-dependent changes in metabolites pre-exercise, during, and post-exercise and to provide complete data on the metabolic regulation of exercise and aging. 190, 197, and 187 metabolites changed respectively with time in C, A, and AE groups were monitored (Table S2). Recent research indicated that aging appeared to have a stronger effect on the blood metabolome than an exercise in rats,^31^ which was also confirmed in this paper. The changed metabolites of the aging group were more redundant than exercise.

Not surprisingly, aging and exercise gave rise to major changes in metabolites related to amino acid metabolism, such as glycine, serine, and threonine metabolism; l-tryptophan metabolism; and l-cysteine metabolism (Figure 6D). The metabolites of the glycine/serine/threonine were elevated with longer leukocyte telomere length (LTL). LTL is a predictor of biological aging; short LTL is associated with an increased risk of age-related disease and death. Exercise training had been shown to reduce the rate of telomere shortening during aging, companied with increased glycine, serine, and threonine metabolites.^32^ Meanwhile, Serra and his colleagues also found that animals with exercise exhibited more metabolites, including glycine,^33^ which mitigated the potentially harmful effects of aging.^31^ Furthermore, exogenous glycine supplementation also improved a variety of aging characteristics.^34^ Glycine was also involved in the metabolism of arginine and bilirubin, in which arginine supplementation significantly attenuated senescence-induced apoptosis,^35^^,^^36^ increased antioxidant activity, and inhibited inflammation^37^ to minimize organism aging.^38^ Bilirubin, an endogenous antioxidant,^39^^,^^40^ levels were inversely associated with the incidence of age-related diseases.^41^ Appropriate exercise increased the level of bilirubin in the body and provided more benefits for older people.^42^ Interestingly, serine is involved in the TCA cycle and cysteine metabolism. Aging is associated with oxidant/antioxidant imbalances in the body, including a reduction in cysteine concentration.^43^ Acidic and cysteine-rich proteins declined with age, which were induced by exercise and had positive effects on aging-related metabolic diseases.^44^^,^^45^ Supplementation with N-acetylcysteine and glycine extended lifespan, ameliorated multiple age-related deficits,^34^^,^^46^ and even reversed multiple age-related abnormalities to enhance physical health in the elderly.^47^ In this paper, glycine, serine, arginine, and cysteine levels increased over time after the 14th day in all groups, yet the overall levels in group C and AE were higher than those in group A (Figure 6C). About bilirubin, group A showed a continuous declining trend while group C and AE began to rise respectively in the 14th and 28th day, resulting in higher expression than group A (Figure 6C). The results suggested that exercise might retard aging by upregulating the levels of glycine, serine, arginine, bilirubin, and cysteine. It was worth mentioning that *cis*-aconitate, the metabolites of the TCA cycle, were monitored and the changed trend was almost consistent in all three groups. *Cis*-aconitate decrease continued with time but turned to increase after the end of the exercise. The level of that in group A was consistently higher than that in the other two groups during the decline period (Figure 6C), which might be due to the aconitase. The activity of aconitase decreased most dramatically with age among the enzymes involved in the TCA cycle,^48^^,^^49^ resulting in the accumulation of *cis*-aconitate in aging individuals. The relatively subtle changes in TCA circulatory enzyme activity were sufficient to cause an overall decrease in the efficiency of mitochondrial bioenergetics.^48^^,^^49^ Therefore, we speculated that exercise might improve the metabolic pathway of D-gal-induced aging individuals by regulating TCA enzyme activity.

It was believed that only L-amino acids were involved in protein synthesis. However, in living organisms, free D-amino acids and several peptides containing D-amino acids were isolated. Many neuropeptides isolated from organisms contain D-isomers in their chains, and the number was increasing. More importantly, these neuropeptides had stronger biological activity compared to L-amino acids.^50^ In this study, D-amino acids were also detected, including D-ornithine and diaminopimelic acid involved in D-amino acid metabolism. After exercise intervention, the levels of two metabolites were close to those of young individuals (Figure 6C), indicating that D-gal disordered D-amino acid-related metabolism, which recovered balance through exercise.

Different from amino acids synthesized in the body, tryptophan can only be obtained from diet. Systemic and cellular tryptophan levels were determined by dietary intake and the activity of the pathways that convert or degrade tryptophan, more than 95% of which was degraded through the kynurenine pathway.^51^ The ratio of kynurenine to tryptophan, which reflected the tryptophan degradation rate, was elevated in older adults, suggesting that senility was accompanied by accelerated tryptophan degradation via the kynurenine pathway.^52^^,^^53^ However, subsequent studies had shown that aging did not appear to have a major effect on plasma tryptophan concentrations, but its availability increased during exercise.^54^ The availability of tryptophan in the brain was significantly increased during continuous exercise in older men, along with a significant increase in serotonin synthesis and activity, which participated in the antidepressant effect in the elderly.^55^ The benefits of exercise on neuronal integrity and mental health might also imply an increase in kynurenic acid from tryptophan metabolism.^56^ In a recent study, a galantamine-memantine combination was designed for the clinical treatment of Alzheimer’s disease due to the role of reducing tryptophan levels, which meant high concentrations of tryptophan might be involved in the development of Alzheimer’s disease.^57^ In this paper, a higher level of tryptophan was monitored in group A than that in groups C and AE, which might attribute to the reduced metabolism of tryptophan in D-gal-induced aging rats. With exercise, the level of tryptophan was close to that in group C (Figure 6F), which mirrored the finding that exercise enhanced the availability of plasma tryptophan.^54^

Similarly, the expression of 5-hydroxyindoleacetate, one of the metabolites of tryptophan, was higher in group A than that in groups C and AE, suggesting that the level of 5-hydroxyindoleacetate in D-gal-induced aging individuals regulated by exercise was close to that in younger individuals (Figure 6F). In addition, indole-3-pyruvic acid (IPA), a ketone group analog of tryptophan, replenished cells with important biochemicals and behaved as a strong antioxidant.^58^ Patients treated with IPA alleviated anxiety and had a positive effect on mood.^59^ The study monitored a dramatic increase in IPA levels in response to exercise, so it was hypothesized that IPA might be beneficial in delaying D-gal-induced aging through neuroprotection (Figure 6F). Indoleacrylic acid, another indole metabolite, had a similar trend with tryptophan, and those high levels might relate to inflammation.^60^ The higher levels of indoleacrylic acid in group A detected in this paper may be due to inflammation in D-gal-induced aging individuals, while exercise reduced the level to close to that of group C (Figure 6F).

Nutrients in the body included amino acids, vitamins, and others.^61^ The aging individuals were deficient in vitamin B6^62^ but maintained good physical function through the adequate dietary intake of vitamin B6 and other nutrients,^63^ which improved neurocognitive function^64^ and prevented Parkinson’s.^65^^,^^66^ 4-pyridoxic acid (4-PA) was the final excretion of vitamin B6,^67^ whose excretion in urine increased with age.^68^ Exercise steadily increased the concentrations of 4-PA,^69^ which was in agreement with our findings in this paper that vitamin B6 and 4-PA decreased in the three groups at first, reaching the lowest point in the 14th day, and then gradually increased with time (Figure 6E). The levels of vitamin B6 and 4-PA in groups C and AE were higher than those in group A, indicating that exercise made vitamin B6 and 4-PA levels in D-gal-induced senility close to those in adults.

Exercise and aging regulated the levels of amino acids and their derivatives through participation in amino acid metabolism. This article showed that D-gal disordered metabolism in the body, while proper exercise maintained metabolic balance to delay aging in D-gal-induced senile individuals. Taken together, a comprehensive, time-resolved metabolomics analysis about the response of metabolites to D-gal-induced aging and exercise was exhibited in this research. The fact that aging and exercise produce dissimilar metabolites is well known, yet the dynamic changes are frequently disregarded. The dynamic effects of D-gal-induced aging and exercise on metabolites with time were delineated in our data. The translational potential of this work lay in understanding the time-resolved variation of metabolites responding to exercise. Ultimately, enhanced knowledge of exercise and aging can be used to improve interventions on aging.

### Limitations of the study

The main limitation of this paper concerned the number of samples and the proper control. This study lacked a few samples due to the long duration, especially on the 63rd day. Fortunately, the number of surviving samples could still be analyzed because the initial sample size was large enough. The other important limitation of the study was ignoring placing the D-gal-induced and control rats on the treadmill without running.

## STAR★Methods

### Key resources table

**Table:** 

REAGENT or RESOURCE	SOURCE	IDENTIFIER
**Biological samples**		

Rats serum samples	This paper	N/A

**Chemicals, peptides, and recombinant proteins**		

D-gal	Solarbio	Cat# IG0540

**Critical commercial assays**		

MDA Content Assay Kit	Solarbio	Cat# BC0025
SOD Activity Assay Kit	Solarbio	Cat# BC0170

**Experimental models: Organisms/strains**		

Rats: Sprague-Dawley (SD)	Chengdu Dossy Biological Technology Co., Ltd.	Certificate No. SCXK2020-030

**Software and algorithms**		

MetaboAnalyst	iCarbonX Diagnostics (Zhuhai) Company Limited	https://www.metaboanalyst.ca/
Mfuzz	iCarbonX Diagnostics (Zhuhai) Company Limited	R package (Mfuzz)
LMM	iCarbonX Diagnostics (Zhuhai) Company Limited	R package (lme4)
Graphpad Prism 9	Graphpad Software	https://www.graphpad.com/

### Resource availability

#### Lead contact

Further information and requests for resources should be directed to and will be fulfilled by the lead contact, Xue Li (lixue2078@126.com).

#### Materials availability

The study did not generate new unique reagents.

#### Data and code availability

Data

All data have been deposited at the web (https://data.mendeley.com/drafts/y3vtw53983) and are publicly available as of the date of publication. DOIs are listed in the key resources table.

Code

This paper does not report original code.

Other items

Any additional information required to reanalyze the data reported in this paper is available from the lead contact upon request.

### Experimental model and study participant details

A total of 45 male SD rats (8 weeks, body weight of 200 ± 10 g, specific pathogen-free (SPF)) (Certificate No. SCXK2020-030) were purchased from Chengdu Dossy Biological Technology Co., Ltd. (Chengdu, China). All rats were adapted to feeding for one week, with sufficient standard chow and water. 5 rats were housed per cage and maintained at constant temperature (24 ± 2°C) and humidity (45%-60%) under natural light/dark cycle. The rats were randomly divided into three groups depending on treatments including normal control (C) with normal saline treatments, D-gal (Solarbio, Beijing, China) induced aging (A) and D-gal induced aging plus exercise (AE) groups with 15 rats in each group. The rats from the A and AE groups were subjected to subcutaneous injection of D-gal with a daily dose of 100 mg/kg for 6 weeks.^70^ After the injection of D-gal, the rats from AE groups were submitted to aerobic intermittent training on a treadmill (Shanghai Xinruan Information Technology Co., Ltd., Shanghai, China) for 5 days per week and continued for 8 weeks.^71^ Firstly, the rats performed warm-up exercises for 10 min (intensity: 50%-60% of maximal oxygen uptake (VO_2_max), 10 m/min) on a treadmill under 0° slope condition. Secondly, rats ran for 4 min (intensity: 80%-90% of VO_2_max, 20 m/min) under the same condition. Thirdly, rats exercised for 3 min (intensity: 60%-65% of VO_2_max, 12 m/min) under the same condition. The exercise except for the warm-up exercise was repeated 5 times to complete the treadmill exercise for 45 min per day (see table below). The study was approved by the Ethics Committee of the Chengdu Sport University [Grant No. (2022) 1]. Samples were obtained by drawing the blood from tail veins after exercise intervention and stored at -80°C for subsequent use.

**Table:** Schematic of AIT for 45 min


Velocity (m/min)	10	20	12	20	12	20	12	20	12	20	12
Time (min)	10	4	3	4	3	4	3	4	3	4	3

### Method details

#### Morris Water Maze (MWM)

MWM, the primary method of estimating learning and memory ability, might be the most widely used behavioral test for rodent animal cognitive functions, that was used to evaluate the learning and memory ability before and after exercise.^72^^,^^73^ MWM system (Anhui Zhenghua Biological Instrument Co., Ltd., Suixi, China) was consist of swimming pool (diameter 150 cm, height 40 cm) and plate (diameter 10 cm, height 35 cm). The pool was filled with water at a depth of 37 cm and divided into four quadrants. MWM test included the navigation phase (1st-6th days) and spatial exploration phase (7th day). The navigation phase lasted 6 days and trained 4 times per day. Before training, the platform was placed in the fourth quadrant of the circular plane in a pool. The rats were placed facing the wall into the pool. Every training was started at different quadrants. The video system (Panasonic, Kadoma, Japan) automatically recorded the time (< 2 minutes) when the rats found the platform and the swimming path. The rats will be led to the platform if they couldn’t find that in 2 minutes and took rest on the platform for 10-20 seconds before the next experiment. The average value of four training was taken as the learning achievement of the day. The platform was removed after the navigation experiment. For the spatial exploration experiment, rats were put into the water at the fourth quadrant, which swimming path and the times of crossing platform were recorded within 2 minutes to observe the spatial positioning capability of rats.

#### SOD and MDA test

The collected serum was assayed for SOD and MDA expression levels according to the instructions of MDA Content Assay Kit (Solarbio, Beijing, China) and SOD Activity Assay Kit (Solarbio, Beijing, China), respectively.

#### Metabolomics profiling

Metabolomic analysis was performed by iCarbonX (Shenzhen, China).

#### Sample preparation

Samples were taken from the -80°C freezer and thawed on ice. After thawing, the samples were vortexed on Vortex QL-901 (Kylin-Bell, China) for 30 s to mix uniformly. 25 μl of the mixture was taken into a centrifuge tube, then 75 μl of pre-cold methanol/acetonitrile (1:1, V/V, Thermo Fisher Scientific, USA) was added, and vortexed for 30 s to mix. The mixture was sonicated in an ice-water bath for 10 min, then incubated at -20°C for 1 h and centrifuged at 13,000 r/min under 4°C for 15 min. After centrifugation, 75 μl of the supernatant was moved into a new centrifuge tube and drained by Refrigerated CentriVap Concentrator (Labconco, USA). 30 μl of methanol/acetonitrile (1:1, V/V) was added into the centrifuge tube, which was vortexed, sonicated, and centrifuged in accordance with the above experimental conditions. Finally, 25 μl of the supernatant was taken into the lining tube of the sample bottle for detection by mass spectrometry.

#### Ultrahigh performance liquid chromatography coupled to tandem mass spectroscopy (UPLC-MS/MS)

The data of this paper was collected from UPLC-MS/MS (Sciex, USA), which detected conditions as follows.

(1) The UPLC consisted of a chromatographic column and mobile phase. The condition of the column was Waters ACQUITY UPLC BEH Amide 1.7 μm, 2.1 mm × 100 mm. The mobile phase included ultrapure water (A phase, which contained 25 mM ammonium acetate and ammonia) and acetonitrile (B phase). The 2 μl mixture of water and acetonitrile was eluted with 0.5 ml/min of flow rate at 40°C and followed the procedure in below table.

**Table:** The procedure of elution gradient

Time (min)	Ratio (water/acetonitrile, V/V)
0	5: 95 V/V
7	35: 65 V/V
8	60: 40 V/V
9	60: 40 V/V
12	5: 95 V/V
(2) The conditions of MS/MS: The temperature of electron spray ionization was 650°C. The ion spray voltage floating was 5500 V (positive, POS) and -4500 V (negative, NEG), respectively. The declustering potential was 60 V. About ion source gas, the gas1 and gas2 were 60 psi, and curtain gas was 30 psi. The collision-induced ionization parameter was high.

### Quantification and statistical analysis

#### Statistical analysis

The data of rats’ weight, MWM and the SOD and MDA level was analyzed by one-way ANOVA analysis of Graphpad Prism 9 (Software, Inc., USA). Results of MWM, SOD and MDA were expressed as mean ± standard deviation. The result of rats’ weight was expressed as mean ± standard error.

This project used the self-developed metabolomics analysis platform to analyze the mass spectrum data statistically.

(1) Preprocessing of metabolic data

The positive and negative ions in the metabolic data were preprocessed respectively. Retained the characteristic of MS2.score > 0.5. When multiple features correspond to the same metabolite name, the highest value was chosen as the value of the metabolite.

(1) Screening for time-dependent metabolic molecules

Mfuzz is a soft clustering approach for time series data, which has a lot of advantages compared with hard clustering. In this paper, for samples within the same group, all metabolite molecules were clustered using the median value for each metabolite molecule at the same time point. The Mfuzz was analyzed by software named R package Mfuzz, which specific procedure were as follows: (1) For each feature, the median values for all samples of the feature at the same sampling time point were calculated. (2) The optimal number of clusters was evaluated based on the minimum centroid distance using the Mfuzz::Dmin() method. (3) Under the current optimal number of clusters, the clustering results were obtained by using the Mfuzz::mfuzz() method. The software named R package pheatmap was used to obtain the membership value of each feature in each cluster based on the clustering results of Mfuzz. The filtered features of each cluster with membership value > 0.7 were standardized, which were draw to heatmaps with the pheatmap () method.

The linear mixed model incorporates random effects into the model compared with the general linear model. In this paper, for samples within the same group, considered differences between and treated the individuals as a random effect, time as variable, and metabolite molecules as dependent variables to screen the metabolites with time-dependent change (*P* < 0.05).
