# Supplementary material for: Time-resolved map of serum metabolome profiling in D-galactose-induced aging rats with exercise intervention
Source: iScience. 2024 Jan 26;27(2):108999. doi: 10.1016/j.isci.2024.108999 (PMC10867647; doi:10.1016/j.isci.2024.108999)
Supplement: Document S1. Figures S1–S7 and Tables S1–S3 [file mmc1.pdf]

iScience, Volume *27*

## Supplemental information

# Time-resolved map of serum metabolome profiling in D-galactose-induced aging rats with exercise intervention

**Xue Li, Changling Wei, Yu Jin, Jinmei Zhang, Pei Zhong, Deman Zhang, and Xiaohan Huang**

**SUPPLEMENTAL INFORMATION**

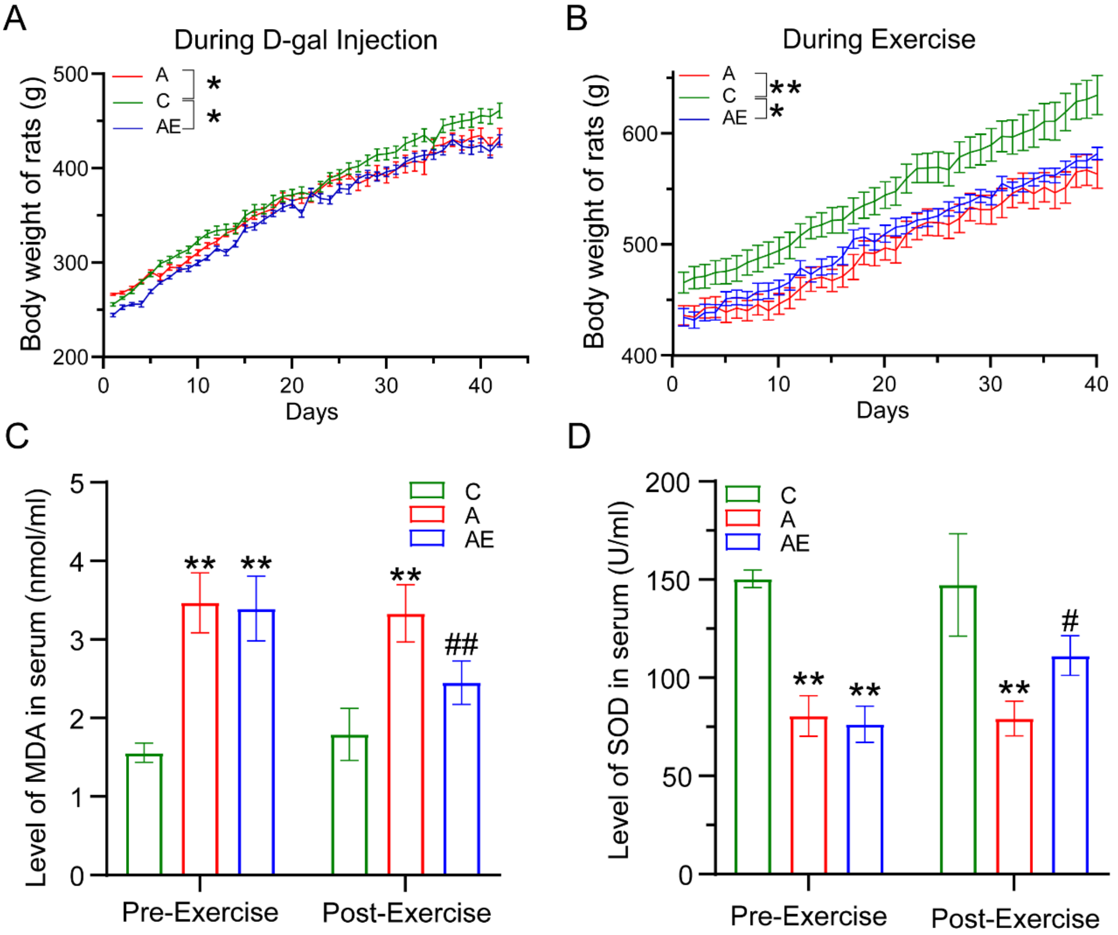


Figure S1 The body weight of rats and the level of malondialdehyde (MDA) and superoxide dismutase (SOD) in serum of rats. Related to Figure 1.

(A) and (B) The body weight of rats during D-gal injection and exercise, respectively (n > 12, Data was represented as mean ± standard error and was analyzed by one-way ANOVA analysis of Graphpad Prism 9. * $P < 0.05$ and ** $P < 0.01$ indicate significant difference compared with C group, respectively).

(C)and (D) The level of MDA and SOD in serum of rats before and after exercise. (n = 5, Data was represented as mean ± standard deviation was analyzed by one-way ANOVA analysis of Graphpad Prism 9. * $P < 0.05$ and ** $P < 0.01$ indicate significant difference compared with C group, respectively; ## $P < 0.01$ and # $P < 0.05$ indicate significant difference compared with A group).

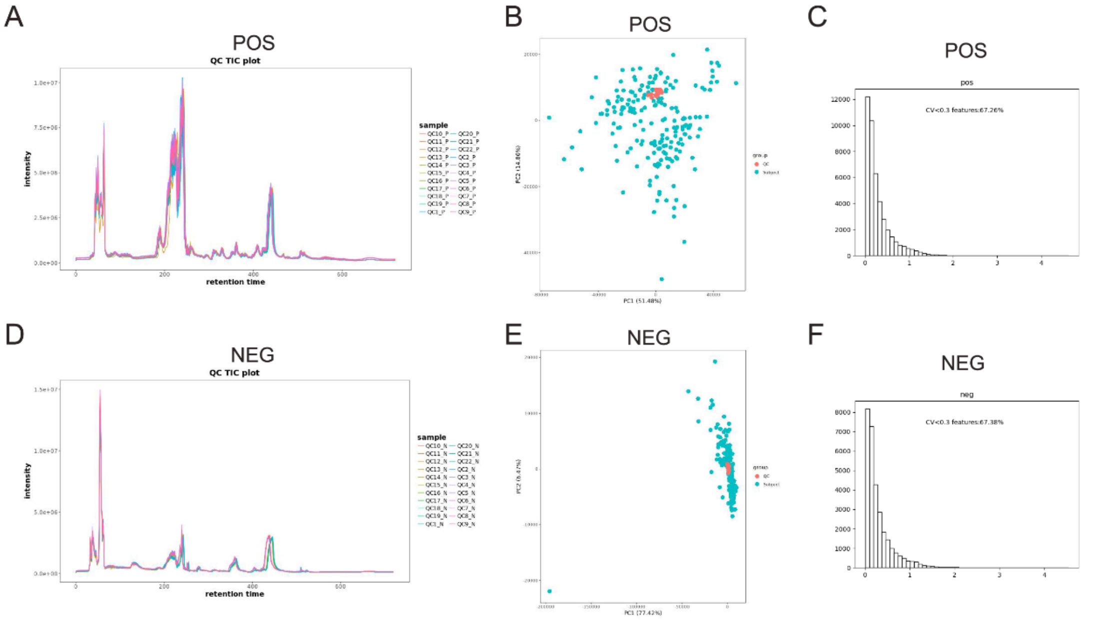


Figure S2 The quality control of data. Related to Figure 2.

(A) and (D) The total ion chromatography (TIC) overlap diagram of quality control (QC) sample.

(B) and (E) The principal-component analysis (PCA) of QC sample.

(C) and (F) The coefficient of variation (CV) distribution map. ((A), (B) and (C) were in positive ion mode; (D), (E) and (F) were in negative ion mode).

The QC were the same samples, which TIC overlap diagram can be used to determine the state of the instrument. The higher degree of the overlap indicates that the instrument is more stable. The higher aggregation of QC samples means that the instrument is more stable and the quality of data is better. CV value reflects the stability of the eigenvalue. The smaller CV value indicates that the eigenvalue is more stable.

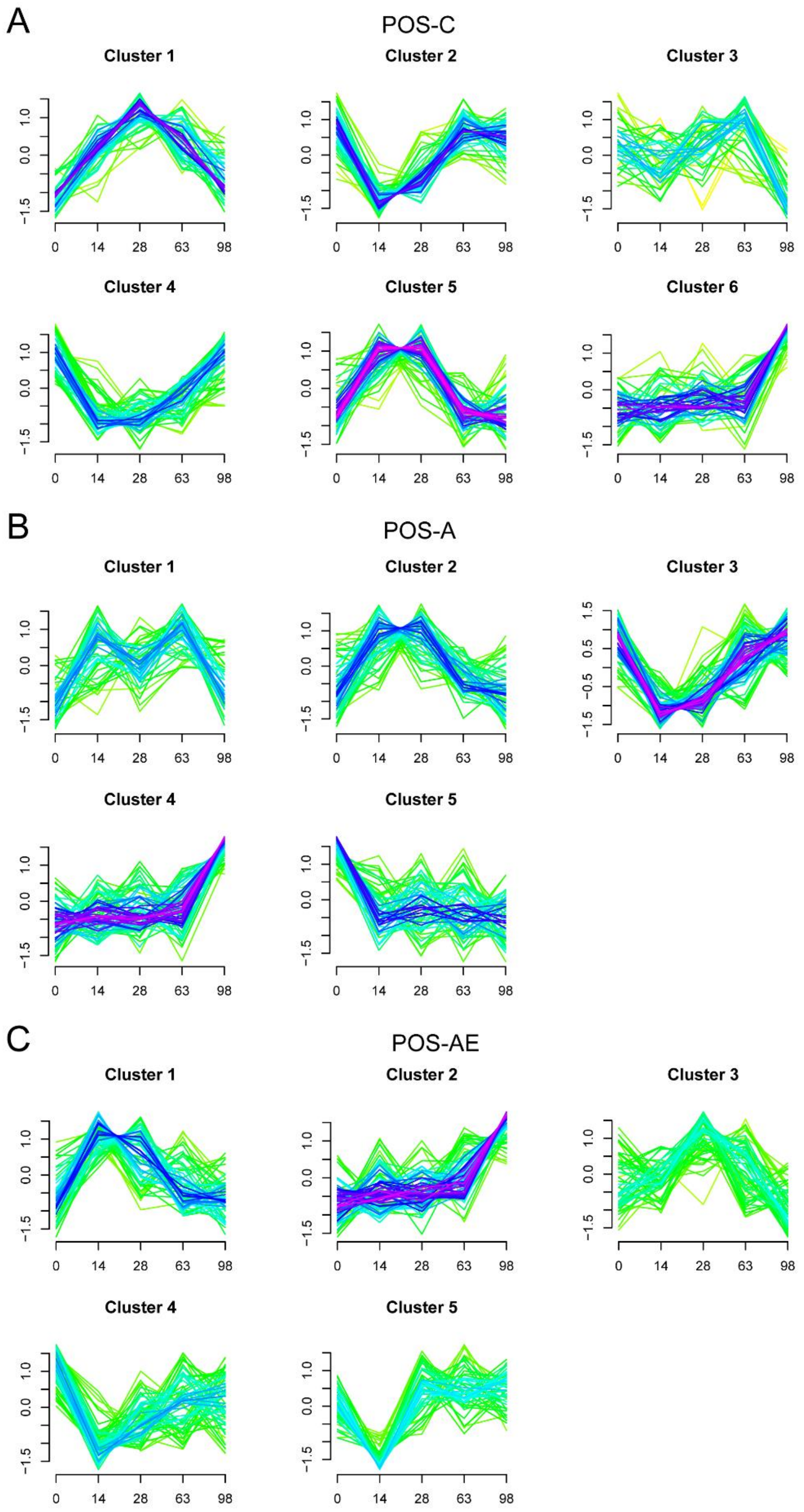


Figure S3 Clusters of changes in positive metabolite with time. Related to Figure 3.

(A) The clusters of metabolites in Group C.

(B) The clusters of metabolites in Group A.

(C) The clusters of metabolites in Group AE. The vertical coordinate is the value of the metabolite after scale treatment and the horizontal coordinate is time (days).

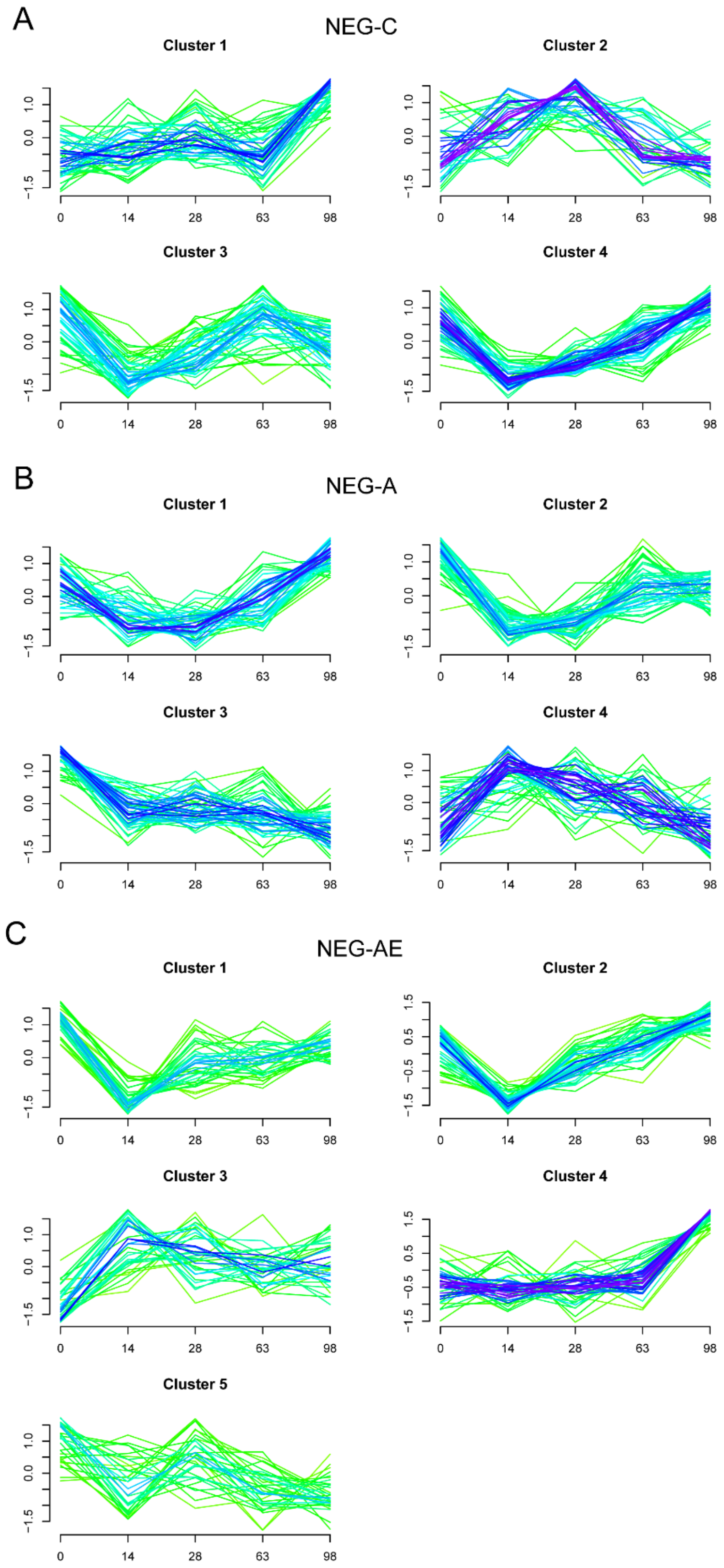


Figure S4 Clusters of changes in negative metabolite with time. Related to Figure 4.

(A) The clusters of metabolites in Group C.

(B) The clusters of metabolites in Group A.

(C) The clusters of metabolites in Group AE. The vertical coordinate is the value of the metabolite after scale treatment and the horizontal coordinate is time (days).

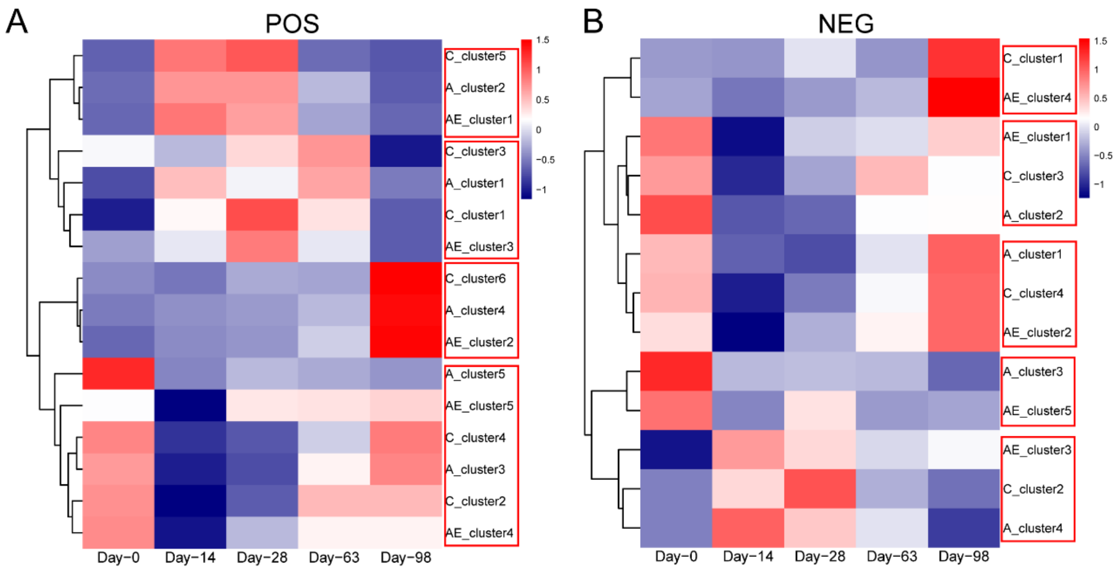


Figure S5 Patterns analysis. Related to Figure 5.

(A) The 16 clusters of positive metabolite were clustered into 4 patterns.

(B) The 13 clusters of negative metabolite were clustered into 5 patterns.

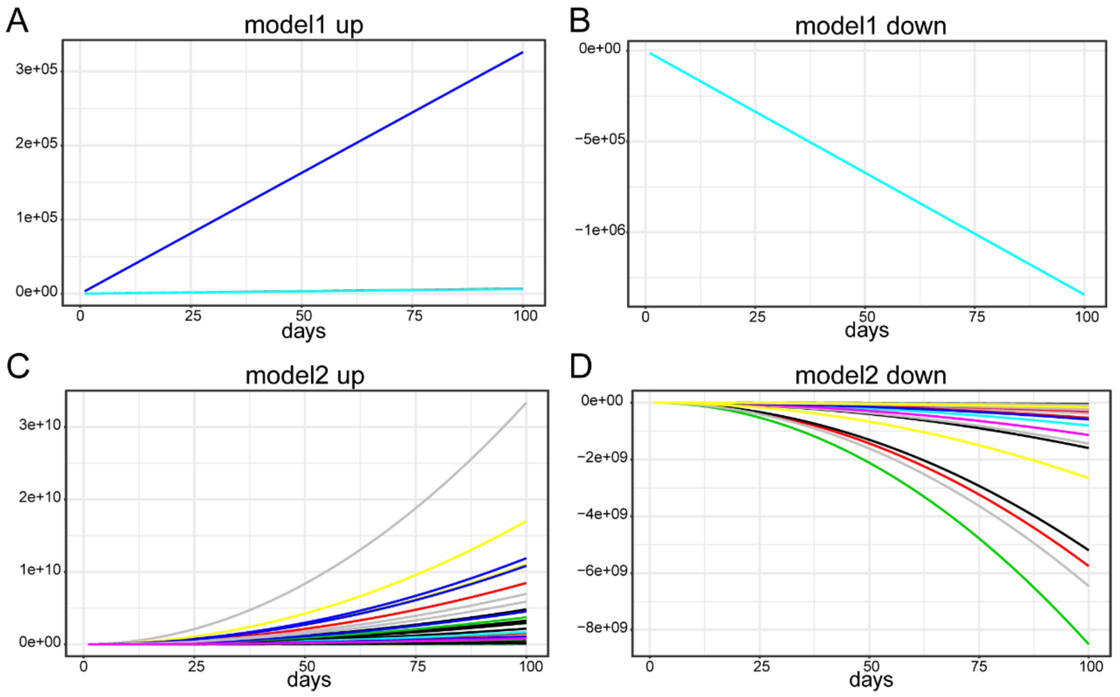


Figure S6 The classification of patterns. Related to Figure 6.

Model1 up indicates that the variable is a first-order function and the coefficient is positive. Model2 up indicates that the variable is a second-order function and the coefficient of the quadratic term is positive. In the same way, model1 down indicates that the coefficient of the

first-order function is negative. Model2 down indicates that the coefficient of the quadratic term in the second-order function is negative.

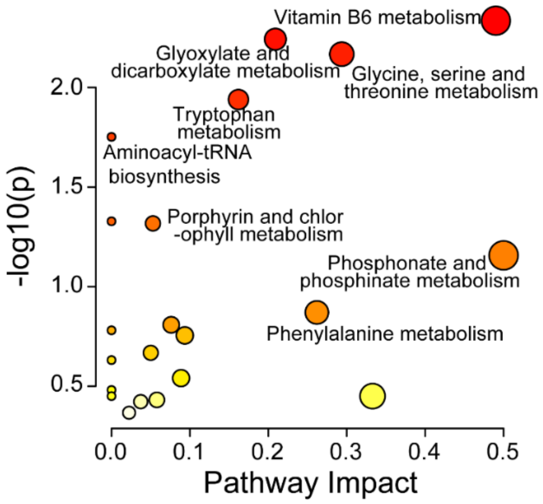


Figure S7 Bubble plot of metabolic pathway analysis with metabolites related to aging obtained via Kyoto Encyclopedia of Genes and Genomes (KEGG) analysis. Related to Figure 6.

**Table S1 Statistics of changes in core metabolites between different groups Related to Figure 5.**

| | Comparison | Change | The number of metabolites |
|---|---|---|---|
| POS | C to A | Changed pattern | 6 |
| | | Invariable pattern, score up | 26 |
| | | Invariable pattern, score down | 32 |
| | A to AE | Changed pattern | 10 |
| | | Invariable pattern, score up | 12 |
| | | Invariable pattern, score down | 25 |
| NEG | C to A | Changed pattern | 7 |
| | | Invariable pattern, score up | 10 |
| | | Invariable pattern, score down | 8 |
| | A to AE | Changed pattern | 14 |
| | | Invariable pattern, score down | 10 |

**Table S2 Statistics of metabolites changed significantly over time in each group with the four types of models (including positive and negative ions). Related to Figure 6.**

| Model | Group C | Group A | Group AE |
|---|---|---|---|

| | | | |
|---|---|---|---|
| Model1 up | 21 | 15 | 25 |
| Model1 down | 19 | 26 | 22 |
| Model2 up | 82 | 110 | 77 |
| Model2 down | 68 | 46 | 63 |

**Table S3 The list of 31 selected metabolites. Related to Figure 6.**

| ID | Group C | Group A | Group AE |
|---|---|---|---|
| Arg-Glu | model2_down | model1_up | model2_down |
| 3-Methylhistamine | model2_down | -- | model2_down |
| 4-Pyridoxic acid | model2_up | -- | mode1_up |
| Asn-Gln-Arg | model1_down | -- | model1_down |
| Indole-3-pyruvic acid | model2_down | -- | model2_down |
| Indoleacrylic acid | model2_down | -- | model2_down |
| L-Cystine | model2_up | -- | mode2_up |
| Cysteine-S-sulfate | model2_down | -- | model2_down |
| 1,2-Benzenedicarboxylic acid | -- | model2_down | -- |
| Diaminopimelic acid | -- | model1_down | -- |
| Ephedrine | -- | model2_up | -- |
| Formylanthranilic acid | -- | model2_up | -- |
| Glyceric acid | -- | model1_down | -- |
| Pyridoxal | -- | model2_up | -- |
| 1,2-Di-(9Z-octadecenoyl)-sn-glycero-3-phosphocholine | -- | model2_up | -- |
| 5-Hydroxyindoleacetate | -- | model2_down | -- |
| L-Tryptophan | -- | model2_down | -- |
| Nicotinate | -- | model1_down | -- |
| Pro-Lys-Arg | -- | model2_up | -- |
| Allantoin | model2_down | model1_down | model2_down |
| 1-Stearoyl-sn-glycerol 3-phosphocholine | model1_up | -- | mode1_up |
| Arachidonic Acid (peroxide free) | model2_up | -- | model2_up |
| Bilirubin | model2_up | -- | model2_up |
| cis-Aconitate | model2_up | -- | model2_u |
| Thymine | model2_down | -- | model1_down |
| Phenylpyruvate | model2_up | -- | model2_up |
| D-Ornithine | -- | model2_up | -- |
| DL-Serine | -- | model2_up | -- |
| Glycine | -- | model2_up | -- |
| L-Arginine | -- | model2_up | -- |
| cis-9-Palmitoleic acid | -- | model1_down | -- |
